# Experimental Evaluation of PSC Structures from FRP with a Prestressing Strengthening Method

**DOI:** 10.3390/ma14051265

**Published:** 2021-03-07

**Authors:** Tae-Kyun Kim, Sang-Hyun Kim, Jong-Sup Park, Hee-Beom Park

**Affiliations:** Department of Infrastructure Safety Research, Korea Institute of Civil Engineering and Building Technology, 283, Goyang-daero, Ilsanseo-gu, Goyang-si 10223, Gyeonggi-do, Korea; kimsanghyun@kict.re.kr (S.-H.K.); jSpark1@kict.re.kr (J.-S.P.); heebeompark@kict.re.kr (H.-B.P.)

**Keywords:** prestressed concrete, fiber-reinforced polymer, strengthening method, near surface mounted, external prestressing, advanced composite materials

## Abstract

A prestressed concrete (PSC) structure is subject to prestress losses in the long and short terms, and the structure ages over time. The structure is susceptible to corrosion from exposure to environmental factors such as moisture, chloride, and carbonation, thus causing prestress loss. Therefore, strengthening the structure is needed to address this problem. Here, the near surface mounted (NSM) method and the external prestressing (EP) method were selected because they are capable of applying additional prestressing. Further, we used fiber-reinforced plastics or polymers, or carbon fiber-reinforced plastics or polymers because of their high tensile strength and noncorrosive properties. For EP tests, prestressed strands were used. Accordingly, this study performs four-point flexural tests and evaluations for 12 types of specimens fabricated with different PSC methods. All specimens fabricated with the NSM (prestressing, no prestressing) and EP methods achieved stiffness that was 50–60% higher than that of the control PSC specimen. It was observed that the EP method in conjunction with prestressing yielded the best strengthening effect. It is expected that the results of this study will be applied to real structures for strengthening them and improving their performances.

## 1. Introduction

Following the shortage of steel in Europe during World War II, prestressed concrete (PSC) structures developed at a rapid pace. According to the design, the concrete in existing reinforced concrete structures withstands compression, and the steel bar withstands tension. However, owing to the nature of the constructure, efficient use of materials is difficult; durability is reduced with larger structures and the development of cracks. Thus, PSC was introduced to overcome these limitations [1]. In recent years, PSC structures have gained worldwide attention because of their economic feasibility, ease of construction, esthetics, safety, convenience of maintenance, and harmony with nature and the environment; they are extensively applied in the construction of infrastructure [1]. However, PSC structures are subject to short- (elastic deformation, friction, and anchorage activity) and long-term (creep, relaxation, and dry shrinkage) prestress losses after the prestress force is applied [1,2].

In case of short-term loss, when a prestress force is applied to a PS member, elastic deformation occurs to the extent of the compressive force received by the member [3]. In addition, the behaviors of the sheath pipe and PS steel are not completely in consonance, resulting in friction between them; accordingly, when a prestress force is applied to the PS steel, the PS steel anchorage slips owing to the force [4,5]. In case of long-term loss, creep causes deformation owing to the continuous stress generated during the curing process. Additionally, dry shrinkage occurs when moisture inside the concrete evaporates, thus reducing its volume [6,7,8,9]. Regarding relaxation, after the prestress force is applied, the prestressing of the PS steel relaxes and then plateaus over time. These various types of PS losses have a direct and indirect impact on the structures [10,11,12,13]. In fact, PSC structures such as bridges, nuclear power plants, and roads show rapid deterioration with aging, leading to the possibility of property damage and casualties. Therefore, strengthening of the structure is required to enforce measures against deteriorating aged PSC structures.

In civil engineering, strengthening refers to the restoration or the improvement of the load-carrying capacity of a structure to the level of the original design. Various strengthening methods are used for strengthening concrete structures, such as near surface mounted (NSM), external prestressing (EP), external bonding (EB), section enlargement (SE), and steel plate adhesive strengthening. Each method is implemented by considering the location and environment of the structure [14,15,16,17,18,19,20,21,22,23]. Numerous studies have been conducted with the EB and SE strengthening methods in real structures. Examples include (a) research on the strengthening of structures by the SE method according to the quantities of the reinforced beams, and the associated evaluation [24]; (b) evaluating the strengthening effect of reinforcing concrete structures with steel plates, carbon fiber sheets, and reinforced beams [25]; (c) efficacy comparison of NSM and externally bonded reinforcement (EBR) techniques based on grooves according to the number of layers of reinforcements, and the number of grooves [26]; (d) evaluation of the performance of the EB method when used under multilayer fiber-reinforced plastic or polymer (FRP) sheets for flexural strengthening of concrete beams [27]; and (e) performance evaluation of varying number of applied bonded carbon fiber composite laminates according to the length of the strengthening application [28].

However, in the case of NSM and EP methods, a clear and unified design had not been presented until recently, with only a few studies using these methods. Nevertheless, active research on design methods according to various strengthening methods is being pursued at the American Concrete Institute (ACI) and in Europe; the results have been established into relevant guidelines and manuals. However, in South Korea, design research efforts in relation to strengthening methods have been limited [29]. Furthermore, following the adoption of the NSM method early on, the strengthening of structures has been achieved mainly using reinforced bars. However, performance evaluations of prestressed NSM carbon fiber-reinforced polymer (CFRP) systems [30] and EBR combined with the NSM technique for CFRP sheets [31] have been recently conducted [30,31]. Additional research is required in this field.

Therefore, to assess the NSM method with FRP in detail, we installed an anchorage, and CFRP was used as the reinforcement material. Further, we selected the NSM and EP methods and conducted comparative analyses with the unmodified PSC structure as the control. In the NSM method, grooves were constructed in the structure, and various appropriate reinforced materials were laid into the grooves. When prestressing was required, the anchorage was also placed. After the placement of the reinforced materials, high-strength epoxy or grout was used as the groove filler material for bonding, as first performed by Asplund [32]. Through external prestressing, this method has distinct advantages, as it allows improvements in the stress condition of the existing member, prevents sagging, and controls cracks.

The FRP used herein is a composite material mainly reinforced with glass or carbon fiber. Its properties include corrosion resistance, high strength, and light weight; it can serve as an alternative to reinforced bars. CFRP is produced by adding carbon fiber to plastic to increase its strength and elasticity. This yields a higher tensile strength, lower weight, significantly lower density, and smaller coefficient of thermal expansion than those of iron. It has drawn recent attention as a lightweight material for use in the interior and exterior parts of automobile, industrial, construction, and sports goods [33,34].

This study examines the behavioral characteristics of structures according to various strengthening methods after applying additional prestressing. In this experiment, additional strengthening was applied to different test specimens with a given internal prestress force. This differs from the design strategy that considers strengthening the specimen in the process of fabrication. In addition, when additional prestressing is applied to the structure, information such as the internal prestress loss cannot be accurately obtained from the reinforced structure. Further, there may be practical problems such as the detachment of the anchorage after strengthening. Therefore, it is expected that this study will contribute toward establishing the basis for a design strategy to impart improved strength to concrete materials.

## 2. Experimental Materials and Structural Test Details

### 2.1. Mixture Properties

The properties of the concrete mixtures are listed in Table 1. The low and high design strengths are 20 and 40 MPa, respectively. This strength setting is based on the fact that old structures were mainly constructed with general-strength concrete, although recent structures mostly use high-strength concrete. In the cement mixture, ordinary Portland cement (OPC), water, sand, gravel, fly ash, air-entraining agents, and a high-strength ground granulated blast-furnace slag (GGBS) of 40 MPa were used. The chemical composition of fly ash was 57SiO_2_-25Al_2_O_3_-10Fe_2_O_3_-3CaO-1SO_3_, and the chemical composition of GGBS was 45CaO-35SiO_2_-13Al_2_O_3_-5MgO.

### 2.2. Test Specimen Design and Fabrication

**Figure 1:**
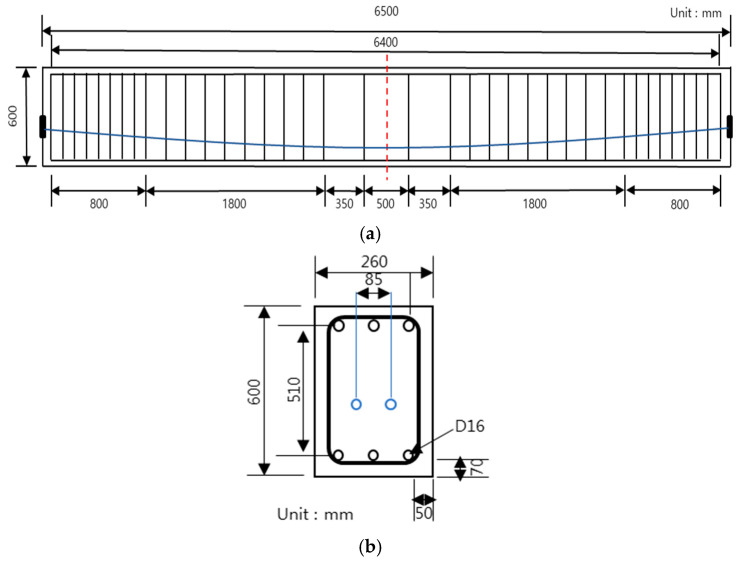
Specimen type and detail: (**a**) prestressed concrete (PSC) specimen and (**b**) cross-sectional details.

**Figure 2:**
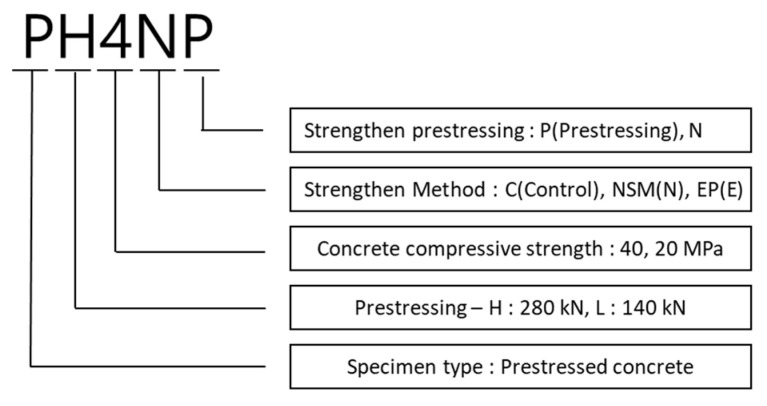
Explanation for the nomenclature of specimen codes.

**Figure 3:**
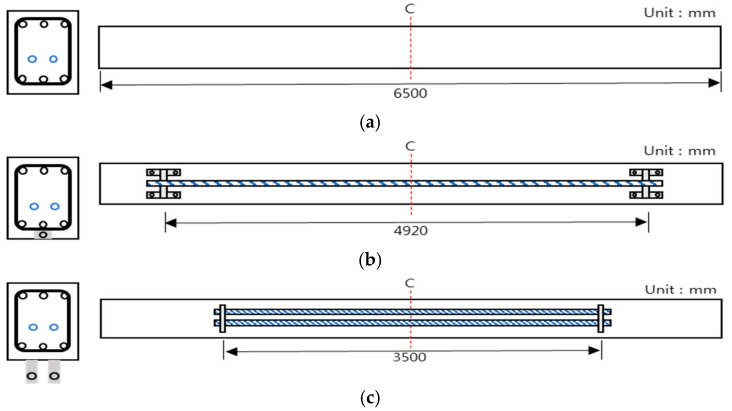
Specimen strengthening methods: (**a**) control, (**b**) near surface mounted (NSM), and (**c**) external prestressing (EP).

Figure 1 shows schematics of the test specimen details and its cross-section. The basic specimen has a PSC structure even without any application of strengthening. The specimen is 6500 mm long (L), 600 mm high (H), and 3200 mm in length from the center to the end. D-16 steel bars were used for both the compressive and tensile steel bars of the specimen, where three bars were placed for each type, and D-10 was used for tie bars. The material for specimen prestressing was located 257 mm from the lower end, and two SWPC 7 B PS strands (diameters: 12.7 mm) were used. Figure 2 and Table 2 show the descriptions of the specimen types, and Figure 3 shows the details of the strengthening method. There were 12 specimens in total, and only one specimen was tested under varying prestressing forces, different strengthening methods, and different strengthening materials. The specimen was a single PSC structure with a default value of 280 kN used for prestressing. This study performed a comparative analysis of strengthening with the aging of the structure, considering that prestressing was reduced over time that resulted in 50% long-term loss. The compressive strengths of concrete were set to 20 and 40 MPa, and the type of strengthening method was categorized as the control PSC specimen and NSM and EP method cases. In the case of the reinforced material, CFRP was used for the NSM method and PS strands were used for the EP method. Further classification was conducted depending on the prestressing status of the reinforced material. Table 3 shows the material properties of the steel bars, strands, and CFRP bars. The length of the strengthening application in Figure 3 is 4920 mm for NSM and 3500 mm for EP, and anchorage was used.

### 2.3. Test Method

**Figure 4:**
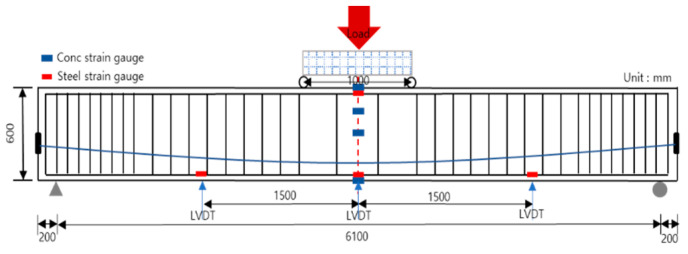
Flexural test setup (using a concrete strain gauge, steel strain gauge, and a linear variable differential transformer).

Figure 4 shows the experimental setup for the flexural test of the PSC structure. Thus, the test method is based on KS F 2408 (standard tests for the flexural strength of concrete), but the researcher can select the test method according to their needs and test environment [35]. The test on the PSC specimen was performed 28 days after concrete curing, and the loading test was performed with a 2000 kN universal testing machine. In general, the types of concrete curing consist of dry, wet, and steam curing. In this study, all the test specimens were subjected to dry curing to resemble the environment of the actual construction site. Load control was performed at a speed of 0.03 mm/s for displacements up to the initial 30 mm, and displacement control was performed at a speed of 0.1 mm/s thereafter. Regarding the reaction point, hinged and roller supports were installed at 200 mm from both ends. For the gauge used in this study, strain was measured using a concrete strain gauge (Tokyo Sokki Kenkyujo, Tokyo, Japan), steel strain gauge (Tokyo Sokki Kenkyujo, Tokyo, Japan), and linear variable differential transformer (LVDT, Tokyo Sokki Kenkyujo, Tokyo, Japan). The LVDT was installed at the center of the specimen and at the L/4 points on both sides, the steel strain gauge was installed at the tensile steel bar, compressive steel bar, and L/4 of the tensile steel bar, and the concrete strain gauge was attached at the center of the span, upper and lower parts, and at the upper 1/4 point. For the four-point test, the load was applied at points 500 mm from the upper center of the specimen on both sides. In addition, the data logger TDS-530 was used for measurements.

In this study, a comparative analysis was conducted between the design values and the measured flexural test values. The values presented by the manufacturers of the specimens were used as the design values. The crack load (P*_cr_*) was the load applied on the PSC structure associated with the formation of the initial crack, the yield load was the load at which the steel bar yielded (P*_y_*), and the ultimate load was the load at which the structure was finally destroyed (P*_u_*). A comparative analysis of these loads on the specimens was conducted; various analyses were performed on characteristics such as PSC prestressing, concrete strength, strengthening method, and comparison of reinforced material performance according to the manufacturer.

## 3. Experimental Evaluation Results

### 3.1. Load–Displacement Tests

Table 4 compares the design and experimental values for each specimen in terms of the crack load (P*_cr_*), yield load (P*_y_*), and ultimate load (P*_u_*). It can be observed that the maximum and minimum differences between the design and experimental values of the control specimens PH4C, PL4C, PH2C, and PL2C are in the ranges of crack load of −0.31–2.85%, yield load of 10.12–21.40%, and ultimate load of 4.44–9.17%. In the case of NSM (prestressed) specimens PH4NP and PL4NP, the ranges of differences were crack load −1.64 to −5.55%, yield load 7.73–11.27%, and ultimate load −2.91–0.90%. In the case of NSM (no prestressing) specimens PL2NN (H) and PL2NN (S), the ranges of differences were crack load −18.80–25.13%, yield load 12.18–17.64%, and ultimate load 9.56–19.56%. With EP specimens PH4EP, PL4EP, PH2EP, and PL2EP, the ranges of differences were crack load 1.02–25.75%, yield load 12.98–17.56%, and ultimate load 5.35–42.41%. As it can be observed from these results, in terms of the crack load, the NSM (no prestressing) method exhibits a large difference between the design values and the experimental values. In terms of the yield load, the experimental values are higher than the design values, and in terms of the ultimate load, the experimental values are also higher than the design values except for the PL4NP case. However, in the case of PL4NP, the experimental value was not considerably low, but lower by approximately −2%. It is thought that this can be considered to be within the margin of error of the test process. Finally, when comparing the experimental values of the control specimen and each strengthening method, it can be observed that the EP method has 1.5 to 2 times higher crack, yield, and ultimate loads compared with NSM (prestressed or not prestressed states).

### 3.2. Load–Displacement Test Outcomes

**Figure 5:**
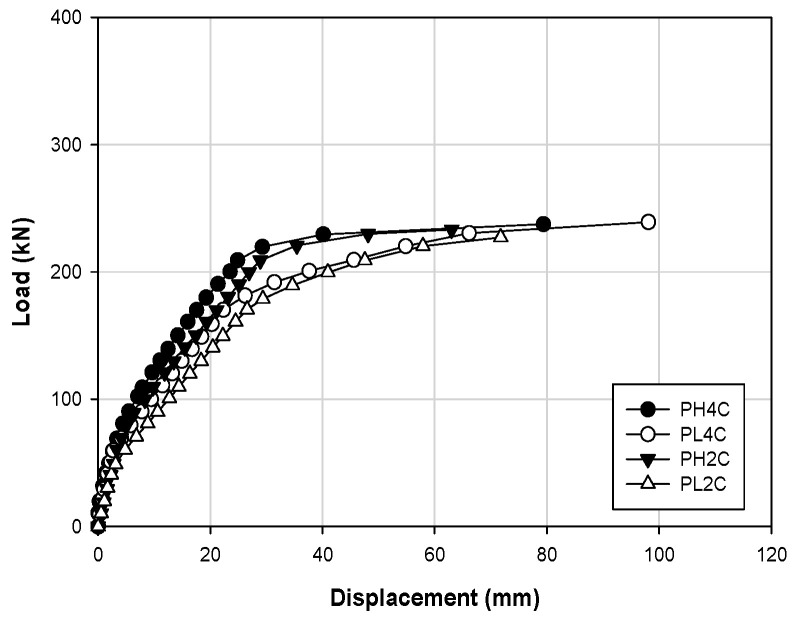
Load–displacement responses upon prestressing of PSC specimens.

Table 4 shows the load–displacement results, and Figure 5 shows the load–displacement curves of the control PSC specimens PH4C, PL4C, PH2C, and PL2C. Regarding the behavior of the structure, PH4C and PH2C yielded similar behaviors, and PL4C and PL2C were similar to each other. Therefore, in the case of the control specimen, the prestress force of the internal prestressing material, rather than the concrete strength, had a considerable impact on the stiffness of the structure. The ultimate loads were 237.6 and 239.3 kN for PH4C and PL4C, respectively, and the displacements were 79.35 and 98.10 mm, respectively. In contrast, PL2C shows the lowest ultimate load and displacement at 227.3 kN and 71.76 mm, respectively.

**Figure 6:**
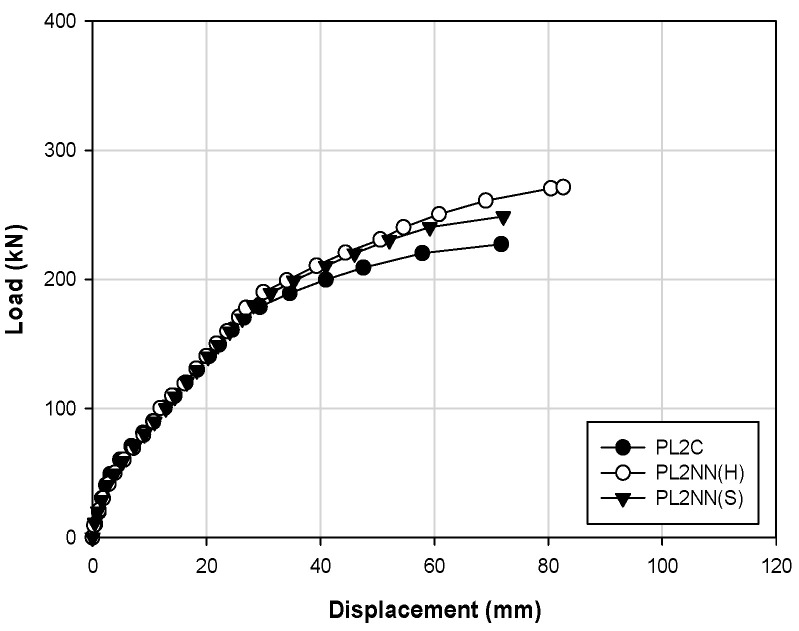
Load–displacement responses for different strengthening materials.

Figure 6 shows the load–displacement curve of the NSM (no prestressing) specimen. In the case of the reinforced material, the performance of products of manufacturers H and S was comparatively analyzed with the control specimen. For specimens PL2C, PL2NN (H), and PL2NN (S), the concrete strength was the same at 20 MPa, and the reinforced material was embedded in one strand obtained from each respective manufacturer. For loads up to the crack load, PL2C, PL2NN (H), and PL2NN (S) showed similar behaviors with load values at 50.6, 46.2, and 42.6 kN, respectively, and displacements equal to 3.57, 3.36, and 2.64 mm, respectively. The yield loads were 180.4, 198.1, and 188.8 kN, respectively, and the corresponding displacements were 30.17, 32.32, and 31.75 mm, respectively. These indicate that the reinforced material of manufacturer H was higher by 8%, but the stiffness shown in the graph is similar. However, from the moment of yielding up to the ultimate load, the loads were 227.3 (PL2C), 271.4 (PL2NN(H)), and 248.7 (PL2NN (S)) kN, respectively, thus indicating that the values of the materials from manufacturers H and S are approximately 20% and 10% higher than that of the control specimen. The maximum displacements were 71.76, 82.64, and 72.12 mm, respectively, and the displacement of the H manufacturer material was larger by approximately 15%. Therefore, in the structural performance evaluation, the result indicates that the material from manufacturer H yielded a superior strengthening effect than the material from manufacturer S.

**Figure 7:**
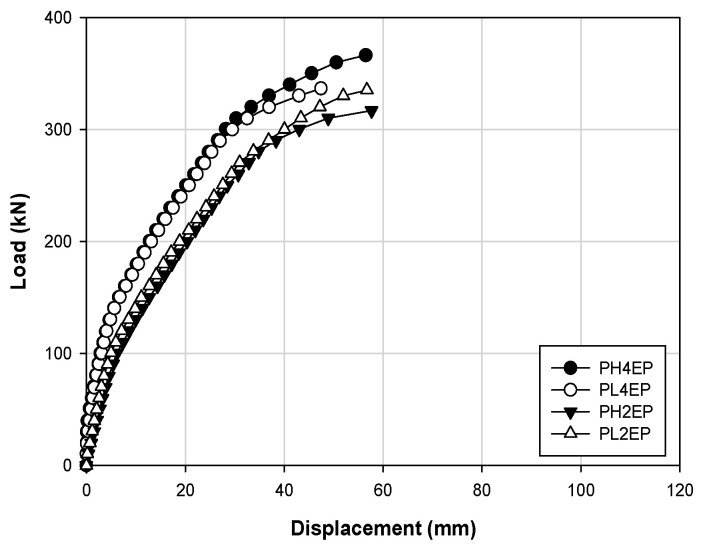
Load–displacement responses for materials with different concrete strengths.

Figure 7 shows the relationship between the concrete strength and strengthening effect in the form of a load–displacement curve and comparatively analyzes the specimens PH4EP, PL4EP, PH2EP, and PL2EP. In the initial stiffness from the crack load to the yield load, the PH4EP and PL4EP specimens show similar trends in behavior, and the PH2EP and PL2EP specimens showed similar behaviors. In addition, the ultimate loads are 356.4 and 336.8 kN for PH4EP and PL4EP, respectively, and the displacements are 56.49 and 47.43 mm, respectively. These outcomes indicate that PH4EP shows higher values, and the ultimate loads are 317.0 and 335.8 kN for PH2EP and PL2EP, respectively. Additionally, the displacements are 57.66 and 56.70 mm, respectively, thus indicating that PL2EP had a higher load value than PH2EP, and the displacement was similar between the two. Furthermore, when comparing PH4EP and PH2EP, and PL4EP and PL2EP, the maximum loads increased by approximately 12.43% and 0.45%, respectively, depending on the concrete strength. Thus, the load has a greater impact on the structural behavior at high strength than at low strength.

**Figure 8:**
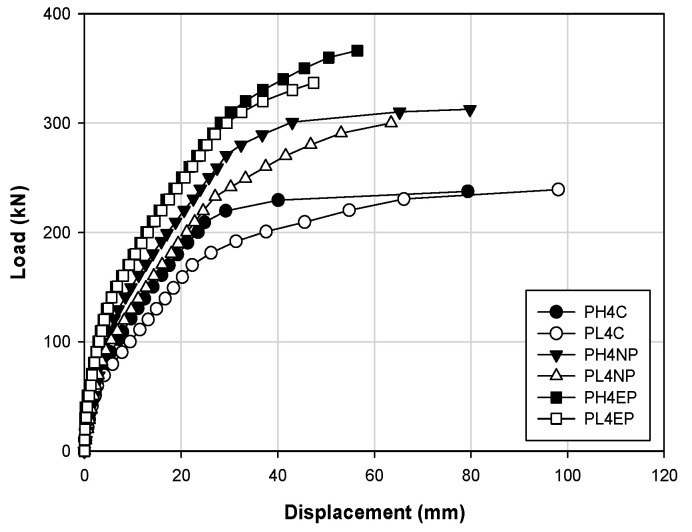
Load–displacement responses for strengthening methods for a concrete strength of 40 MPa for prestressing at 140 and 280 kN.

Figure 8 shows the load–displacement curve according to the strengthening method at the strength of 40 MPa, which corresponds to high-strength concrete, with internal prestressing of the member at 280 and 140 kN. From Figure 8, it can be observed that the EP specimens PH4EP and PL4EP have 50–60% higher stiffness than the control specimen. In addition, the NSM (prestressing) specimens PH4NP and PL4NP have 40–45% higher stiffness than the control specimen. When comparing PH4C and PL4C, PH4NP and PL4NP, and PH4EP and PL4EP, the higher the internal prestressing, the higher the stiffness, given that the other conditions are the same. Owing to the additional prestressing of the reinforced material, the EP and NSM specimens have higher stiffness than the control specimen. However, the structural behavior of the EP specimen exhibits brittle characteristics.

**Figure 9:**
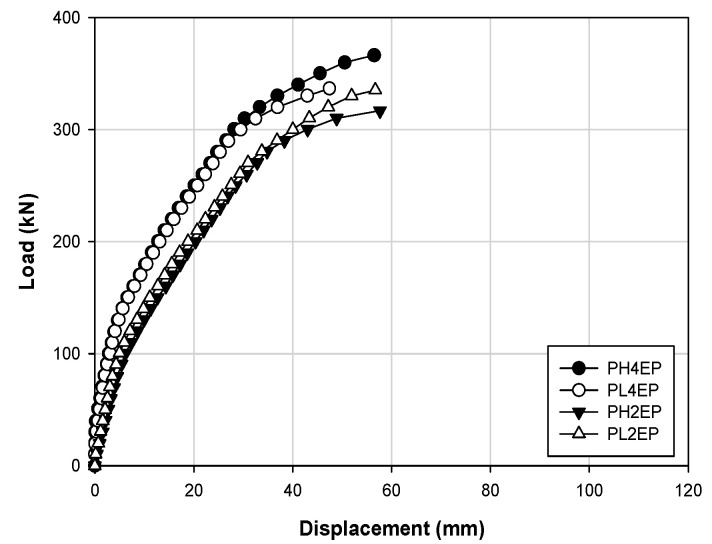
Load–displacement responses for different strengthening methods for a concrete strength of 20 MPa for prestressing at 140 and 280 kN.

Figure 9 shows the load–displacement curve according to the strengthening method for a concrete strength at 20 MPa (low-strength concrete), with internal prestressing of the member at 280 and 140 kN. From Figure 9, it can be observed that the EP specimens PH2EP and PL2EP have 40–60% higher stiffness than the control specimen. The NSM (no prestressing) specimens PL2NN (H) and PL2NN(S) have 5–10% higher stiffness than the control specimen but show similar behaviors to that of the control specimen. When comparing PH2C and PL2C, and PH2EP and PL2EP, the EP specimen PL2EP yields a slightly larger stiffness.

**Figure 10:**
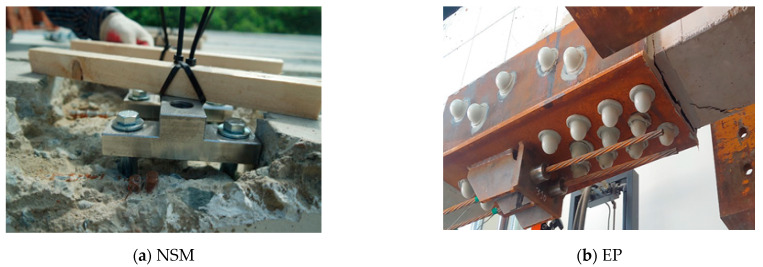
Problems associated with strengthening methods: (**a**) NSM and (**b**) EP.

Figure 8 and Figure 9 show that the stiffness up to the yield load increased in the cases of the PH4EP and PL4EP specimens by nearly twice that of the control specimen. However, in the case of the NSM (no prestressing) specimen, there was no increase in stiffness, similar to the control specimen. From these results, the overall strengthening effect obtained was in the order of EP > NSM (prestressing) > NSM (no prestressing). Furthermore, the higher the stiffness, the more brittle the behavior of the structure. Figure 10 shows concrete strengthening in actual applications according to the proposed methods in this study, where a gap was generated between the anchorage and the grooving when the NSM method was applied. With the application of the EP method, cracks occurred at the anchorage. Therefore, it is thought that EP and NSM methods will show improved strengthening effects when these practical problems are resolved.

### 3.3. Concrete Strain Measurements

**Figure 11:**
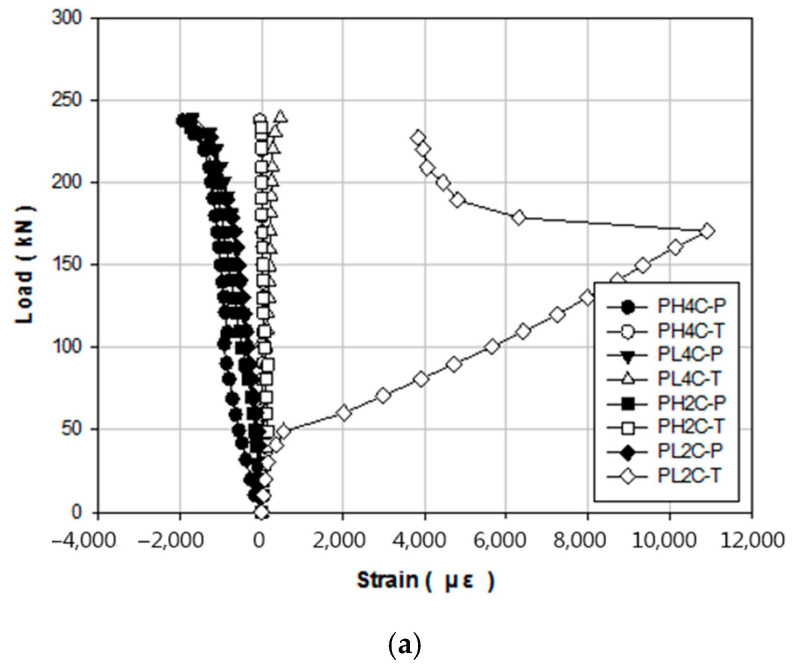

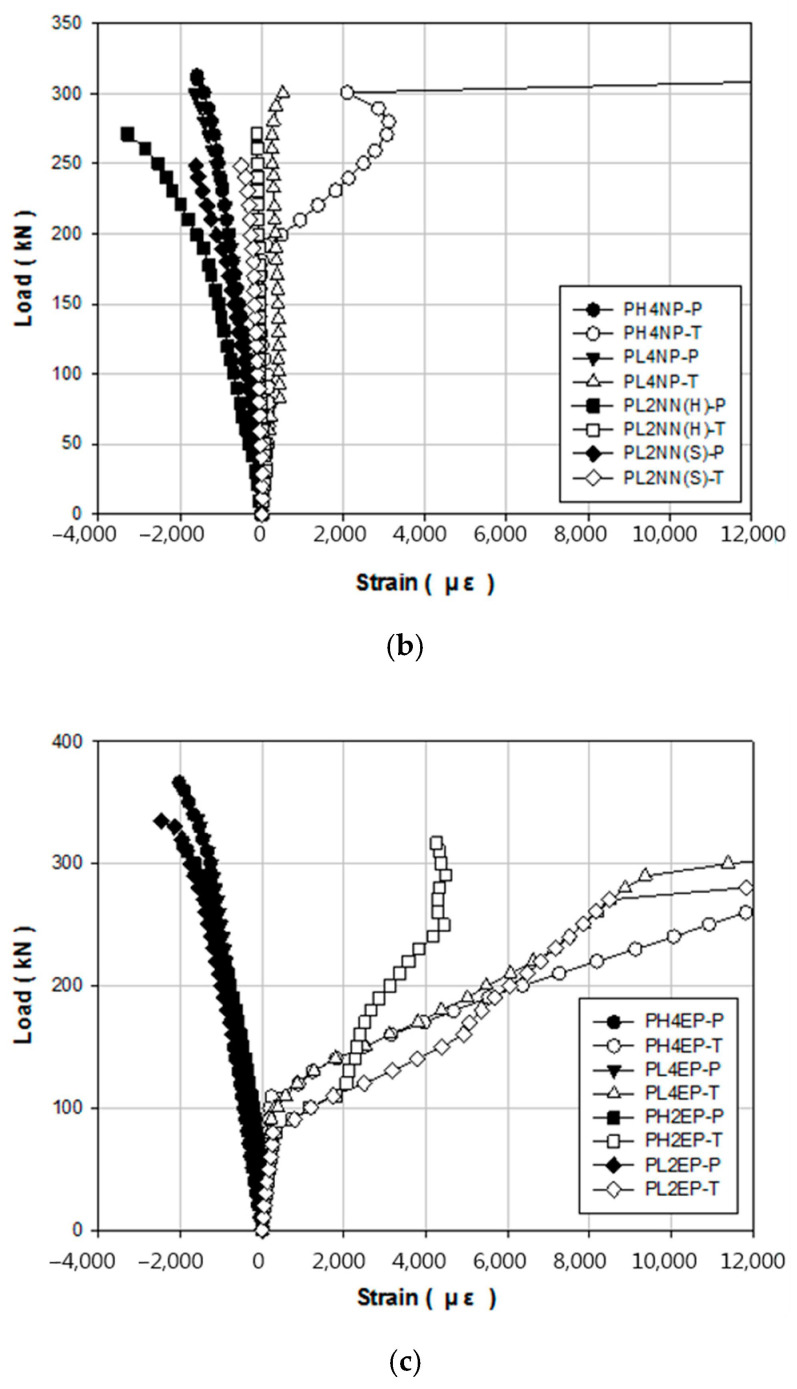
Concrete load–strain responses for different strengthening methods: (**a**) PSC (control), (**b**) NSM, and (**c**) EP.

Figure 11 shows the load–strain on the compression and tension sides of the concrete for each strengthening method. Figure 11a shows the response curve of the control specimen, Figure 11b is the corresponding curve for the NSM (prestressing) specimen and NSM (no prestressing), and Figure 11c is the curve for the EP specimen. For concrete, the compression failure strain was 0.003. As a result of compression failure, the entire member suddenly experiences brittle failure after the concrete undergoes compression, thus indicating that concrete compression failure possesses a considerable risk. As shown in Figure 11, the control, EP, and NSM (prestressing) specimens all reached the ultimate load before they reached the concrete compressive failure strain of 0.003. Thus, it is thought that under the actual experimental ultimate load, there is a structural resistance at the compression edge. However, the concrete compression failure strain of manufacturer H of the NSM (no prestressing) specimen exceeded 0.003. As shown in the load–displacement graph, this phenomenon is thought to have occurred because of inadequate installation of the anchorage after grooving during the concrete strengthening process.

### 3.4. Steel Strain Measurements

**Figure 12:**
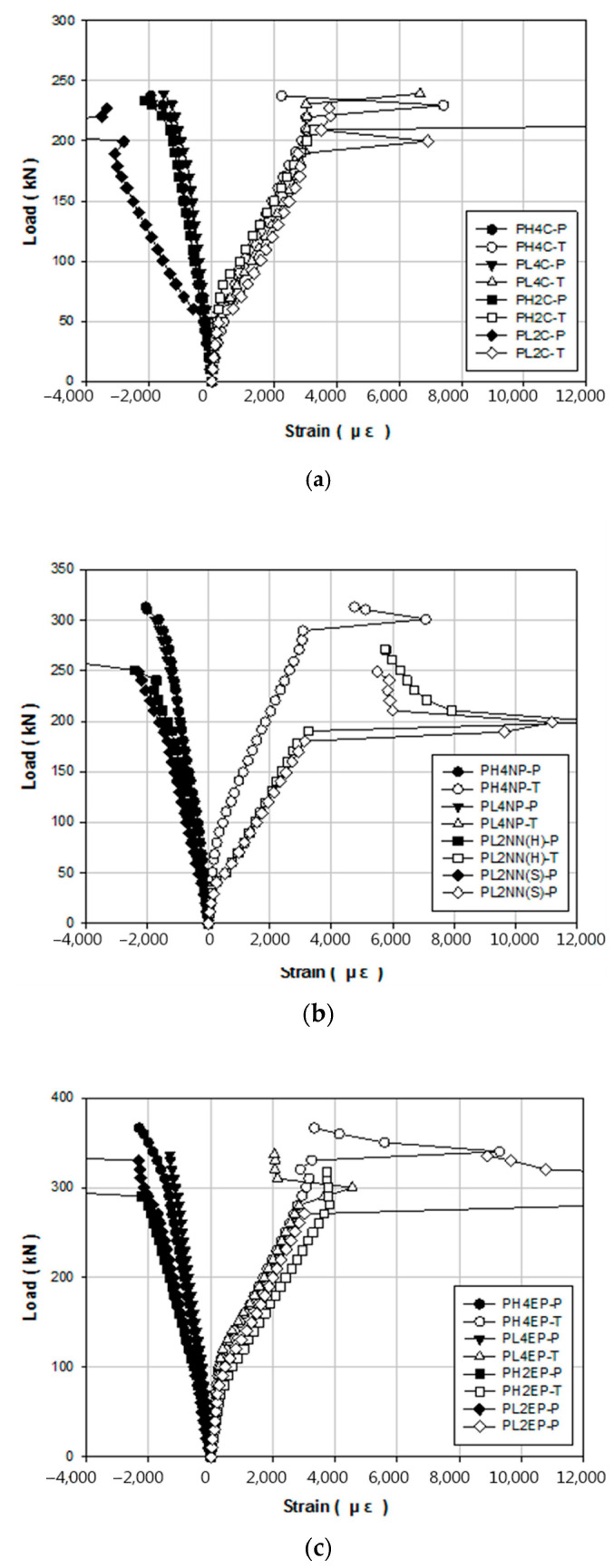
Reinforced bar load–strain responses for different strengthening methods: (**a**) PSC (control), (**b**) NSM, and (**c**) EP.

Figure 12 shows the load–strain responses on the compression and tension sides of the reinforced bar for each strengthening method. Figure 12a shows the curve of the control specimen, Figure 12b is the curve for the NSM (prestressing and no prestressing) specimens, and Figure 12c is the curve for the EP specimen. Notably, because the reinforced bar used in this study has a yield strength of 400 MPa, it is predicted to yield when the strain of the reinforced bar will be approximately 0.002 or higher. In Figure 12a, the control specimen yields at approximately 200 kN. In the case of the NSM (prestressing) specimen (Figure 12b) and the EP specimen (Figure 12c), the maximum load is increased up to approximately 300 kN owing to the strengthening effect. However, it can be observed that the NSM (no prestressing) specimen in Figure 12b has a yield point similar to that of the control specimen.

## 4. Conclusions

In this study, the structural performance was comparatively analyzed according to a variety of variables, such as concrete strength, prestress force, strengthening method, reinforced material, and prestressing status of the reinforced material, to improve the structural performance deterioration that may arise with the aging of PSC structures, and to establish a database for future reinforcement design processes. The results can be summarized as follows:

(1) When comparing the design and the experimental values, the crack load showed a large difference in the NSM (no prestressing) method, but in terms of yield load, all experimental values were higher than the design values. In addition, the EP method values were 1.5 to 2 times higher than the NSM (prestressing or no prestressing) method for each load.
(2) Both the EP and NSM (prestressing) methods applied to high-strength (40 MPa) and low-strength (20 MPa) concrete showed 5% to 60% higher stiffness than that of the control specimen. As the internal prestress force increased, the stiffness increased. Owing to the additional prestressing of the reinforced material according to the strengthening method, the specimens of EP and NSM methods showed higher stiffness than the control specimen, but a brittle behavior was observed for these specimens.
(3) When the load–displacement curves of all specimens were compared, the stiffness up to the yield load increased almost two times compared with that of the control specimen in the cases of PH4EP and PL4EP, but in the case of specimens tested with the NSM method (no prestressing), there was no increase in stiffness and the overall trend was similar to that of the control specimen (strengthening effect: EP > NSM (prestressing) > NSM (no prestressing).
(4) The strain of the reinforced bar was 0.002 or higher, and both the NSM (prestressing) and EP methods showed a higher yield strength than the control specimen owing to the strengthening effect. However, the NSM (no prestressing) method yielded a strengthening effect similar to that of the control specimen. It is thought that in the process of applying the NSM method, the gap between the anchorage and concrete led to a decrease in the strengthening effect.
(5) Among the various strengthening methods discussed in this study, the EP method had problems at the interface between the anchorage and concrete, and the NSM method had problems with the gap between the anchorage and concrete, and with the integrated behavior of members. Furthermore, other common problems of different material characteristics were also noted depending on the manufacturer of the reinforced material. To address these problems, further investigations using a variety of strengthening methods, amounts of reinforced materials, and reinforced materials from different manufacturers are required.

## Figures and Tables

**Table 1:** Design strengths of concrete mixture constituents.

Design Strength (MPa)	Unit Weight (kg/m^3^)						
	OPC	W	G	S	FA	GGBS	AE
20	265	162	905	954	30	-	2.36
40	258	151	1039	626	77	180	5.37

Abbreviations: OPC, ordinary Portland cement; W, water; G, gravel; S, sand; FA, fly ash; GGBS, ground granulated blast-furnace slag; AE, air-entraining agent.

**Table 2:** Description of test specimens (the codification of specimens is explained in Figure 2).

No.	Specimen	Specimen Prestressing (kN)	Strengthening Method	Strengthening Material	Strengthening Amount (ea)	Strengthening Prestressing
1	PH4C	280	-	-		-
2	PL4C	140	-	-		-
3	PH2C	280	-	-		-
4	PL2C	140	-	-		-
5	PH4NP	280	near surface mounted (NSM)	carbon fiber-reinforced plastic or polymer (CFRP)	1	prestressing
6	PL4NP	140	NSM	CFRP	1	prestressing
7	PL2NN (H)	140	NSM	CFRP	1	-
8	PL2NN (S)	140	NSM	CFRP	1	-
9	PH4EP	280	external prestressing (EP)	steel	2	prestressing
10	PL4EP	140	EP	steel	2	prestressing
11	PH2EP	280	EP	steel	2	prestressing
12	PL2EP	140	EP	steel	2	prestressing

**Table 3:** Material properties of steel, carbon fiber-reinforced polymer (CFRP), and strands.

Material Property	Steel Bar	CFRP Bar	Strands
Young’s modulus (GPa)	200	165	200
yield stress (MPa)	400	-	1597.9
ultimate stress (MPa)	560	2750	1880.7

**Table 4:** Comparison of design and experimental outcomes of crack, yield, and ultimate loads.

No.	Specimens	Crack Load (P*_cr_*) (kN)			Yield Load (P*_y_*) (kN)			Ultimate Load (P*_u_*) (kN)		
		Design *	Experimental	Displacement (mm)	Design *	Experimental	Displacement (mm)	Design *	Experimental	Displacement (mm)
1	PH4C	96.0	94.5	5.90	198.7	218.8	28.14	227.5	237.6	79.35
2	PL4C	63.9	63.7	3.14	152.8	185.5	28.98	227.5	239.3	98.10
3	PH2C	81.3	83.3	5.58	194.8	219.6	33.29	213.7	233.3	63.03
4	PL2C	49.2	50.6	3.57	150.4	180.4	30.17	213.7	227.3	71.76
5	PH4NP	127.7	125.6	6.60	253.7	273.3	28.89	310.0	312.8	79.80
6	PL4NP	95.5	90.2	4.29	208.5	232.0	27.01	309.0	300.0	63.48
7	PL2NN(H)	56.9	46.2	3.36	168.4	198.1	32.32	227.0	271.4	82.64
8	PL2NN(S)	56.9	42.6	2.64	168.3	188.8	31.75	227.0	248.7	72.12
9	PH4EP	125.7	141.7	5.62	283.5	323.9	33.30	326.5	356.4	56.49
10	PL4EP	114.2	132.1	4.95	264.8	311.3	31.72	236.5	336.8	47.43
11	PH2EP	97.6	98.6	6.04	261.9	295.9	37.16	300.9	317.0	57.66
12	PL2EP	86.2	108.4	5.81	244.1	285.8	36.91	300.9	335.3	56.70

* Elastic design theory (non-consideration of bond failure).

## Data Availability

The data presented in this study are available on request from the corresponding author.
